# Endovascular Treatment for Primary Tumor Hemorrhage in Oropharyngeal Carcinoma: A Report of Two Cases

**DOI:** 10.7759/cureus.60483

**Published:** 2024-05-17

**Authors:** Misato Hirano, Yukiyoshi Mita, Jumpei Fukui, Kana Matsushima, Toshimitsu Nemoto

**Affiliations:** 1 Otolaryngology - Head and Neck Surgery, Japanese Red Cross Narita Hospital, Narita, JPN

**Keywords:** interventional radiology, endovascular procedures, acute bleeding, carotid blowout syndrome, oropharyngeal carcinoma, primary tumor bleeding, head and neck cancer, acute tumor hemorrhage

## Abstract

Acute arterial hemorrhage is a damaging and sometimes lethal complication that occurs in patients with head and neck cancer. However, achieving hemostasis can be challenging because of the difficulty in applying pressure in the throat and oral cavity. In this context, endovascular treatment (ET) has been performed in recent years. This report aims to describe the benefits of ET for acute bleeding. Additionally, our findings emphasize the importance of early diagnosis and treatment of tumor-related bleeding, not only for immediate life-saving benefits but also for the potential resumption of irradiation and chemotherapy, which can lead to favorable long-term prognoses in some instances. We describe two cases of primary tumor bleeding where treatment was successful with ET. Neurosurgeons performed these treatments, and effective hemostasis was achieved in both cases. No complications or rebleeding were observed. ET is a better option for hemorrhage from oropharyngeal tumors than for hemorrhage from the main trunk of the carotid artery. The efficacy of ET is dependent on the vessels involved, and early identification of the culprit artery can predict the prognosis. ET should be considered an option for acute arterial hemorrhage in head and neck cancer.

## Introduction

This article was previously presented as a meeting abstract at The 32nd Annual Meeting of the Japan Society for Head and Neck Surgery on January 19, 2023.

Acute arterial hemorrhage is a damaging and sometimes lethal complication that occurs in patients with head and neck cancer. However, it is challenging to achieve hemostasis due to difficulties in manual compression in the throat and oral cavity [1,2]. Endovascular treatment (ET) has advanced in recent years, but there are few reports of acute arterial hemorrhage treated with ET. Here, we describe two cases of primary tumor bleeding successfully treated with ET.

## Case presentation

Case 1

Case 1 was a 77-year-old man with oropharyngeal carcinoma (cT4aN1M0 p16-positive) and a history of hypertension and diabetes mellitus. A tracheotomy was performed and a single course of irradiation (70 Gy/35 Fr) was administered. Subsequently, he was referred and admitted to our hospital. Since his Eastern Cooperative Oncology Group (ECOG) performance status had improved (from ECOG 3 to ECOG 2), we considered adjuvant chemotherapy.

**Figure 1:**
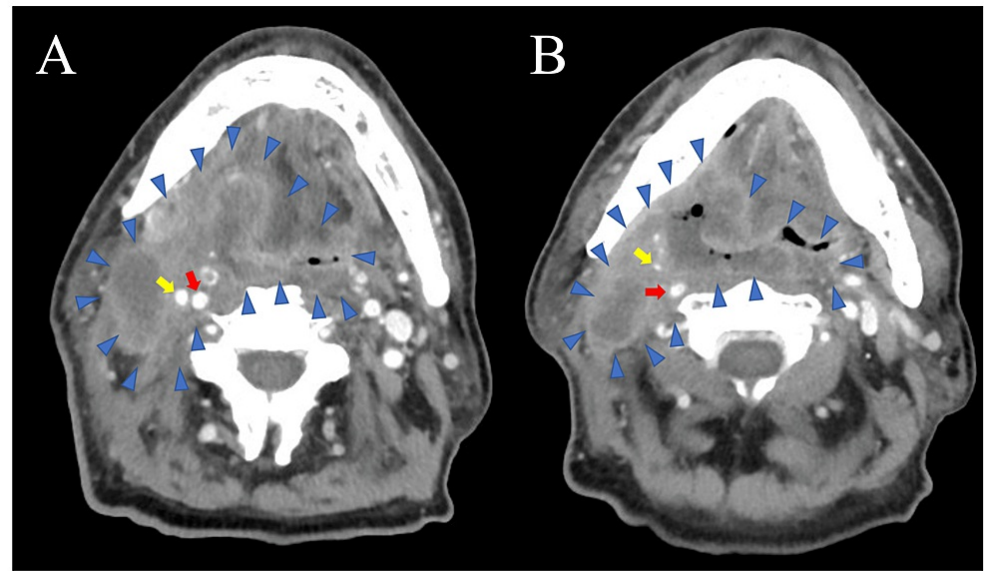
Contrast-enhanced computed tomography in Case 1. (A) Primary tumor enlargement near the right ECA (yellow arrow), and the center of the tumor shows hypodensity. (B) Tumor invasion of the right ECA (yellow arrow). The tumor is surrounded by blue arrowheads. Red arrows indicate the internal carotid arteries and yellow arrows indicate the ECA ECA: external carotid artery

The primary tumor was located in the right oropharyngeal sidewall in contact with the posterior oropharyngeal wall. The fiberscope and visual examination revealed a necrosis of the tumor surface. The contrast-enhanced computed tomography (CT) (Figures 1A-1B) revealed a primary tumor enlargement near the right external carotid artery (ECA).

**Figure 2:**
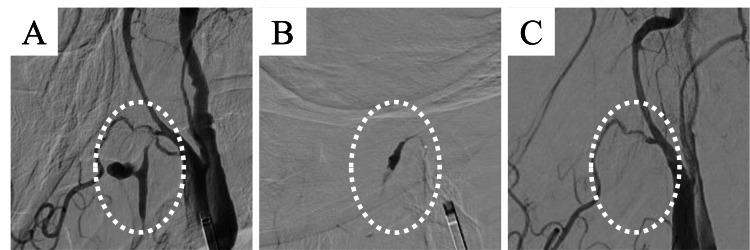
(A) Angiography in Case 1 showing a pseudoaneurysm of the lingual artery branching from the right external carotid artery, with spindle-shaped dilatation and hemorrhage in the surrounding area. (B) n-butyl cyanoacrylate mixed with lipiodol at a ratio of 1:4 is administered to embolize the right lingual artery trunk. (C) External carotid angiography confirms hemostasis.

Twenty-eight days after the completion of irradiation, massive bleeding occurred from the primary tumor, flowing into the oral cavity. The patient’s blood pressure and heart rate were 167/77 mmHg and 111 beats/min, respectively. As hemostasis could not be achieved through manual compression, a neurosurgeon was consulted to conduct ET. Angiography showed a pseudoaneurysm of the lingual artery branching from the right ECA with spindle-shaped dilatation, and hemorrhage was observed in the surrounding area (Figure 2A). Therefore, n-butyl cyanoacrylate mixed with lipiodol at a ratio of 1:4 was administered to embolize the right lingual artery trunk (Figure 2B). The external carotid angiography confirmed hemostasis (Figure 2C).

The patient’s general condition was stable, without rebleeding or cerebral infarction. Chemotherapy (cetuximab 400 → 250 mg/m^2^, carboplatin area under the curve = 2.5 mg/mL·min, paclitaxel 100 mg/m^2^) was started on postoperative day 5. No more tumor bleeding was observed. One month later, the patient died of cancer.

Case 2

Case 2 was a 66-year-old man with oropharyngeal carcinoma (cT4aN1M0 P16-negative) with a history of hypertension, dyslipidemia, reflux esophagitis, cerebral infarction, and emphysema. The patient had a social history of smoking 15 cigarettes/day and drinking one alcoholic drink/day.

Concurrent chemoradiotherapy (cisplatin 100 mg/m^2^) was started, with the second dose of cisplatin reduced to 80 mg/m^2^ and administered. At 42 days after treatment initiation, laryngeal edema worsened, and emergency tracheotomy was required. The radiation therapy was temporarily interrupted at 44 Gy/22 Fr. The fiberscope and visual examination revealed that the primary tumor on the right side of the base of the tongue was covered with necrotic tissue.

**Figure 3:**
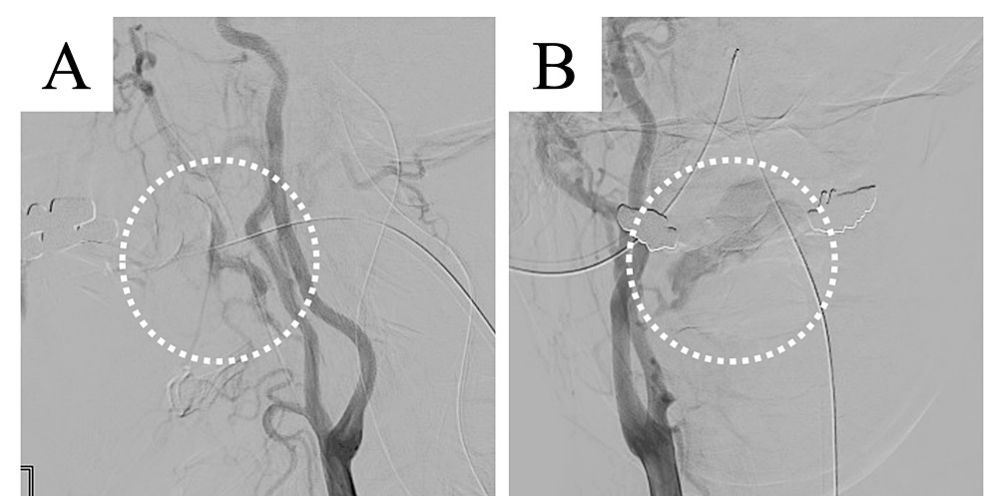
Angiography in Case 2; (A) lateral view and (B) frontal view. External carotid angiography reveals a pseudoaneurysm of the right facial artery. After angiography, the pseudoaneurysm is ruptured and leaks around the right facial artery

Ten days after the emergency tracheotomy, massive bleeding occurred from the primary tumor into the oral cavity. His blood pressure temporarily decreased to 75/52 mmHg. As manual compression could not achieve hemostasis, a neurosurgeon was consulted to conduct ET. External carotid angiography revealed a pseudoaneurysm in the right facial artery. At that time, the pseudoaneurysm ruptured and leaked a contrast agent around the right facial artery (Figures 3A-3B), causing massive bleeding into the oral cavity.

**Figure 4:**
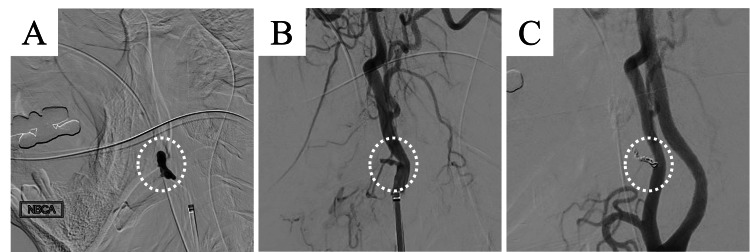
Angiography in Case 2 showing massive bleeding into the oral cavity. (A) The facial artery is embolized using n-butyl cyanoacrylate mixed with lipiodol at a ratio of 1:4. (B) External carotid angiography reveals a blind end in the facial artery. (C) Additional coil embolization is performed at the same site, which successfully achieves hemostasis

The facial artery was embolized using n-butyl cyanoacrylate mixed with Lipiodol at a ratio of 1:4 (Figure 4A). The external carotid angiography revealed a blind end in the facial artery (Figure 4B). The additional coil embolization was performed at the same site, achieving hemostasis (Figure 4C).

The general condition of the patient was stable, without rebleeding or cerebral infarction. Irradiation was resumed on postoperative day 2. The third dose of cisplatin (80 mg/m^2^) was administered on postoperative day 7. He completed 70 Gy/35 Fr of chemoradiation. He experienced a complete treatment response and has been alive for two years and one month after treatment.

## Discussion

In patients with head and neck cancer, acute arterial bleeding is a critical event, with an incidence of 3.9% and a mortality rate exceeding 50% [2]. The conventional treatment for such bleeding is surgical ligation of the carotid artery [3,4]. However, ligature is challenging to perform and involves numerous complications, including a high incidence of cerebrovascular disease. Currently, ET is recommended because it is less invasive [3].

ET of acute arterial hemorrhage has been primarily used in radiology and neurosurgery, and most reports have not specified the responsible vessel or clinical outcomes [1-3, 5-11]. There are few reports of hemorrhage from primary head and neck cancer (Table 1).

**Table 1:** *Reported cases of acute arterial hemorrhage from tumors of the head and neck. *We only included reports that identified the responsible vessels

Author	Number of patients	Type of cancer	Responsible vessels	Incidence of rebleeding (24 hours)	Complications	Treatment received before hemorrhage
Sesterhenn et al. [12]	6	Oropharyngeal cancer (2)	Superior thyroid artery (2)	1	0	Irradiation (6)
							External carotid artery (2)
								Chemotherapy (4)
			Other cancer (4)						Facial artery (2)
						Operation (2)			
							Other arteries (2)		
		Kakizawa et al. [13]						5	Oropharyngeal cancer (2)	Maxillary artery (5)	0	0	Irradiation (2)
														Superior laryngeal artery (2)
						Chemotherapy (2)								
			Lingual artery (2)											
							Other cancer (3)			Operation (1)				
													Facial artery (2)
No therapy (2)													
	Oher artery (1)												

Sesterhenn et al. reported six cases of hemorrhage from head and neck cancer [12], and Kakizawa et al. reported five [13]. Most of these cases involved oropharyngeal cancer similar to ours. Radiation therapy had been performed before bleeding in most cases; therefore, radiation therapy is considered a risk for hemorrhage [14]. In both of our cases, fiberscope and visual examination before bleeding revealed necrotic tissue on the primary tumor surface. This may indicate the extension of necrosis from the tumor interior to the surface, causing massive bleeding. Therefore, we think necrosis of the tumor surface can be a predictive sign of hemorrhage. The recognition of predictive signs will allow prompt CT imaging and time and prophylactic preparation for bleeding, and more reports are awaited to be seen.

Cases of rebleeding are rare [12], probably because the responsible vessels can be completely embolized with ET. There have been no reports of cerebrovascular complications, as the bleeding sites were all branches of the ECA, not the internal or common carotid artery.

Taken together, our findings suggest that ET for arterial hemorrhage of oropharyngeal tumors is highly effective and associated with few complications. However, bleeding from the main carotid artery, referred to as carotid blowout syndrome (CBS), has been reported [2,11]. The ET of CBS involves the risks of rebleeding (9-13%), cerebral infarction (8-14%), and delayed neurological sequelae (10-30%) [1,2,8]. There are no clear conclusions regarding CBS treatment, and therefore, it is essential to determine the source of the bleeding. The leakage from the main carotid artery on angiography is a sign of CBS since the main carotid artery lacks branches.

Early diagnosis and treatment of tumor bleeding, which is a good option for embolization, can save lives and avoid serious complications. The patient may also return to irradiation and chemotherapy after the embolization. A good long-term prognosis can be expected in some cases. For early diagnosis, it is important to carefully examine the patient for predictive signs of bleeding such as necrosis of the tumor surface.

## Conclusions

Acute arterial hemorrhage can be fatal in patients with head and neck cancer. ET for arterial hemorrhage of the primary tumor in head and neck cancer is highly effective and involves few complications. Thus, it should be considered an option for tumor bleeding in head and neck cancer.
